# Deep Wavelet Convolutional Neural Networks for Multimodal Human Activity Recognition Using Wearable Inertial Sensors

**DOI:** 10.3390/s23249721

**Published:** 2023-12-09

**Authors:** Thi Hong Vuong, Tung Doan, Atsuhiro Takasu

**Affiliations:** 1 Department of Informatics, National Institute of Informatics, Tokyo 101-0003, Japan; takasu@nii.ac.jp; 2 Department of Computer Engineering, School of Information and Communication Technology, Hanoi University of Science and Technology, Hanoi 11615, Vietnam; tungdp@soict.hust.edu.vn

**Keywords:** wearable inertial sensors, continuous wavelet transform, multimodal human activity recognition, convolutional neural networks

## Abstract

Recent advances in wearable systems have made inertial sensors, such as accelerometers and gyroscopes, compact, lightweight, multimodal, low-cost, and highly accurate. Wearable inertial sensor-based multimodal human activity recognition (HAR) methods utilize the rich sensing data from embedded multimodal sensors to infer human activities. However, existing HAR approaches either rely on domain knowledge or fail to address the time-frequency dependencies of multimodal sensor signals. In this paper, we propose a novel method called deep wavelet convolutional neural networks (DWCNN) designed to learn features from the time-frequency domain and improve accuracy for multimodal HAR. DWCNN introduces a framework that combines continuous wavelet transforms (CWT) with enhanced deep convolutional neural networks (DCNN) to capture the dependencies of sensing signals in the time-frequency domain, thereby enhancing the feature representation ability for multiple wearable inertial sensor-based HAR tasks. Within the CWT, we further propose an algorithm to estimate the wavelet scale parameter. This helps enhance the performance of CWT when computing the time-frequency representation of the input signals. The output of the CWT then serves as input for the proposed DCNN, which consists of residual blocks for extracting features from different modalities and attention blocks for fusing these features of multimodal signals. We conducted extensive experiments on five benchmark HAR datasets: WISDM, UCI-HAR, Heterogeneous, PAMAP2, and UniMiB SHAR. The experimental results demonstrate the superior performance of the proposed model over existing competitors.

## 1. Introduction

Wearable sensor-based human activity recognition (HAR) plays a significant role in various applications, including sports [1,2,3], smart homes [4,5,6], and purpose-specific monitoring systems [7,8,9]. By extracting information from different sensor signals, such as accelerometers and gyroscopes, a HAR system can accurately recognize various activities, such as running, walking, and sitting. Two common approaches for wearable inertial sensor-based HAR systems are using single modality and multi-modality. A single-modality sensor-based HAR approach [10,11,12,13,14,15] is generally designed to work with a specific type of signal data source, whereas the multi-modality sensor-based HAR approach [16,17,18,19,20,21] processes multiple sensor signals. The single-modality sensor-based HAR methods cannot utilize complementary and comprehensive information from different modalities and only focus on specific tasks, such as fall detection, sitting, and standing. In recent years, multimodal sensor-based HAR methods have become preferable, as they can exploit diverse information from various modalities for various recognition tasks.

Methods for multimodal wearable inertial sensor-based HAR can be further categorized into three groups. The first group, based on a shallow machine learning model, operates in the time domain. Specifically, the methods in this first group split sensor signals into multiple segments using sliding window techniques. These segments are then classified into different action classes using conventional machine learning methods, such as support vector machine (SVM) [16] and random forest (RF) [22]. However, this approach captures only shallow features, often limited by human domain knowledge. Consequently, more discriminative features are not extracted and utilized for more complex activity recognition. Additionally, these methods cannot adapt to other similar activity recognition tasks and involve time-consuming processes to choose optimal features.

The second group, in contrast, employs deep learning (DL) techniques to extract deep features in the time domain. Specifically, convolutional neural networks (CNN) [20,23,24,25,26], hybrid CNN and long short-term memory (LSTM) [19,27,28] are utilized to automatically extract deep features in HAR systems without relying on human domain knowledge. MAG-Res2Net [29] is the latest publication method, which proposed two DL architectures of ResNet [30] with adding a gating module. The MAG-Res2Net model demonstrated robust multimodal performance on two commonly public datasets UCI-HAR [31] and WISDM [32] and leveraged the CSL-SHARE dataset [33]. More precisely, these deep neural network (DNN) methods directly utilize raw sensor signals in the time domain in the form of sequences and extract features through multiple deep neural layers. As a result, the extracted deep features significantly contribute to improving the accuracy of HAR systems. However, this group is constrained within the time domain, which contains limited information about the signals.

Hence, the third group operates in the time-frequency domain by employing transformations through functions like the Fourier transform [18,34,35] and the wavelet transform [21,36,37] to enhance the predictive accuracy of the models. These functions convert raw signals into a spectrum of frequency components, improving the representation of sensor signals compared to using raw sensor data. DNN [17,38,39] are then used to classify each spectrum as an activity. In comparison to wavelet, Fourier transform tends to capture global frequency information over the entire signal. As a result, signal decomposition may not be suitable for all HAR applications, particularly those involving complex activities characterized by short intervals of characteristic oscillation. The wavelet transform, in contrast, can extract local spectral and temporal information simultaneously. Furthermore, it decomposes signals into a set of wavelets, providing a more direct representation of frequency domain distribution in the time domain. With the wavelet transform, both the time and frequency information of the signals are preserved, making it a more powerful transformation for extracting frequency features. In recent publications, as the state-of-the-art results, the wavelet-transform-based and exploring CNNs-based DL methods [21,36,37] are considered, which show promising performance over existing methods in the third group because of several significant reasons. Firstly, these methods used the efficient wavelet-transform-based approach for extracting information from non-stationary signals. Secondly, the methods explored the residual DL architectures of ResNet [30]. The ResNet-based methods address the issue of loss or saturation of accuracy as network depth increases. These methods have drawbacks in complex signal processing and DL architectures. Specifically, the signal processing is complex for suitable wavelet function selection [36] or time-consuming for reducing noise via typical frequency domain filters [37] or requires a well-known residual CNN architecture with 121 trainable layers [21].

In this paper, we introduce a novel model, *deep wavelet convolutional neural networks* (DWCNN), which combines continuous wavelet transform (CWT) and deep convolutional neural network (DCNN) for multimodal HAR using wearable inertial sensors. Our model falls into the category of the third group, employing DNN and operating in the time-frequency domain. However, unlike most methods in this group that use the Fourier transform to compute signal representation in the time-frequency domain, our approach utilizes CWT. Additionally, we propose an algorithm to automatically and adaptively estimate optimal scale parameters for CWT on multiple sensor signals. This facilitates the transformation of these signals into spectrogram images, which then serve as input to the DCNN. The CNN architecture consists of residual and attention blocks, with the former extracting features from different modalities and the latter fusing these multimodal features. We conducted extensive experiments on five benchmark HAR datasets, including WISDM, UCI-HAR, Heterogeneous, PAMAP2, and UniMiB SHAR, to evaluate the performance of the proposed method. The results confirm that our method is more effective compared to existing approaches for multimodal HAR. In summary, the contributions of this paper are as follows:

- Introduction of DWCNN, which is a combination of CWT and DCNN for multimodal HAR.
- Development of an algorithm to automatically and adaptively estimate optimal scale parameters for CWT on multiple sensor signals.
- Designing a DCNN architecture with residual and attention blocks to extract deep features from multimodal signals and classify them into various activity classes.
- Conduction of extensive experiments on five benchmark HAR datasets, including WISDM, UCI-HAR, Heterogeneous, PAMAP2, and UniMiB SHAR, for evaluating performances of the proposed method.

The remainder of this paper is structured as follows: In Section 2, we briefly review existing methods for multimodal HAR. Section 3 outlines the problem definition for multimodal HAR, and Section 4 elaborates on the intricacies of the proposed methodology. Experimental results are reported in Section 5, and Section 6 finally concludes the paper.

## 2. Related Work

Artificial intelligence (AI) and DL are primarily employed in signal processing techniques for HAR. We carefully survey the recent literature on sensor-based HAR using DL in the time domain or time-frequency domain and summarize the findings in Table 1. In the time domain, current research primarily focuses on sensor signal-based HAR using DL techniques. These techniques include CNN [20,23,26,29,40], variants of RNNs like LSTM and GRU, and hybrid DL methods [19,27,28,41,42,43]. Authors such as Yan et al. [40], Cheng et al. [26], and Wang et al. [23] have introduced supporting techniques like the attention layer and convolution layers with various kernel sizes to enhance CNNs for HAR, thereby modifying the original CNN model architectures. Liu et al. [29] proposed MAG-Res2Net, which explored two DL architectures ResNet [30] and added the gated module to improve performance multimodal HAR on three simple and more complex public datasets such as UCI-HAR [31] and WISDM [32] and leveraged the CSL-SHARE dataset [33]. MAG-Res2Net, belonging to the time domain, utilizes raw signals directly without undergoing signal transformation via wavelet transformation. It then feeds these signals into with Res2Net, which integrates multi-scale residual networks and adaptive attention mechanisms. More specifically, MAG-Res2Net employs two continuous ResNet units to explore Res2Net for HAR on the time domain. The accuracy of the MAG-Res2Net method on UCI-HAR, WISDM datasets are 94.26%, 98.42%, respectively. Hybrid models, such as those combining CNN and LSTM or GRU, utilize CNN for spatial feature extraction, LSTM for learning temporal information, and GRU layers for effectively learning long-term dependencies in the data. However, these models typically use raw sensor signals in the time domain and extract features using complex CNN or LSTM architectures, which can be challenging to train and time-consuming. Additionally, some methods are highly specific to particular activity recognition types, as seen in the work of Lee et al. [20]. As a result, these models may not readily adapt to different human activity domains.

Recently, the hybrid wavelet transform and residual CNN-based techniques [21,36,37] were proposed to enhance the accuracy of the multimodal wearable sensor-based HAR problem. In [36], the MLCNNwav model relies on residual CNN and one-dimensional trainable wavelet transform. First, MLCNNwav employs the residual CNN-based architecture to capture global features as the output results. Then, the output of results is fed into a discrete wavelet transform (DWT) to enhance the representation and generalization by learning-activity-related features, whereas PCWCNN [37] first reduces noise via typical frequency domain filter and transform signals by DWT, then uses multilayer residual CNNs to extract features and classification. In [21], the authors proposed CWT-DenseNet, which is the hybrid wavelet transform and DenseNet [44]. The CWT-DenseNet method handles complex with various wavelet functions for each HAR dataset. In addition, CWT-DenseNet explored residual CNN architectures with 121 trainable layers for HAR. However, CWT-DenseNet has the robustness residual CNN-based architecture but only evaluated two small public datasets, specifically the KU-HAR [45] and UCI-HAPT [46] datasets. These methods are time-consuming to select a suitable wavelet function and scale parameters range for different types of signals and the complex residual CNN-based methods.

To address the limitations of existing wearable inertial sensor-based multimodal HAR models, we introduce a novel and robust hybrid model known as the DWCNN model. In Section 4, we will present the problem definition and provide an in-depth exploration of our proposed methodology.

## 3. Problem Definition

In this paper, we tackle the HAR problem using wearable inertial sensors, including accelerometers, gyroscopes, and magnetometers, attached to different positions on each object. Each type of sensor generates three signals, as it measures 3D data along the *x*-axis, *y*-axis, and *z*-axis. To process these data, we employ a channel-based late fusion approach. This approach involves splitting each sensor signal into three input signals based on the channel position. Next, each channel-based signal is further divided into smaller segments using sliding window techniques, enabling us to extract features through network layers and later fuse these features for comprehensive analysis. We represent the raw sensor data input, denoted as S, along with its channels as follows:

(1) $$S=[S_{1},S_{2},\cdots ,S_{i},\cdots ,S_{n_{c}}].$$

Here, $S_{i}=\left(S_{i}^{1},S_{i}^{2},\ldots ,S_{i}^{t}\right)$ for $i\in (1,n_{c})$, where $n_{c}$ represents the number of sensor modalities or input channels, and $S_{i}^{t}$ is the signal vector of the *i*-th channel sensor at time *t*. The goal of the multimodal HAR problem is to split $S_{i}$ into fragments and assign an activity category to each segment. Specifically, given the multimodal sensor data S, our objective is to detect activities within a signal sequence using deep supervised learning techniques in the time-frequency domain.

## 4. Proposed Methodology

**Figure 1:**
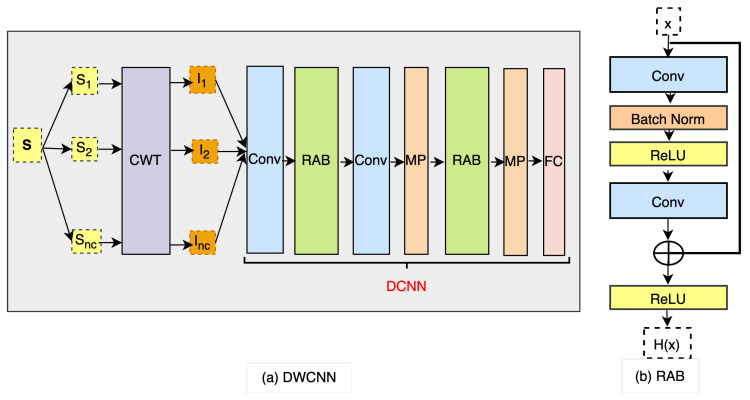
Framework of the proposed DWCNN method.

To facilitate human activity recognition, we introduce the DWCNN model for multimodal HAR using wearable inertial sensors. This model combines the continuous wavelet transform (CWT) with the deep convolutional neural network (DCNN). The framework of the model is depicted in Figure 1. The process begins with the raw sensor input data S, which undergoes a CWT phase. Each signal sequence within $S_{i}$ is transformed into a spectrogram, represented as a CWT image in I${}_{i}$, enabling time-frequency analysis. Subsequently, these CWT images are input into the proposed DCNN, which extracts feature representations for human activity recognition.

### 4.1. CWT

The CWT, as introduced by [47], is a method that transforms a 1D signal x(t), where *t* represents time, into a 2D time-scale representation. This transformation is defined as follows:

(2) $$C\left(a,b\right)={\left|a\right|}^{\frac{-1}{2}}\int_{-\infty }^{\infty }x\left(t\right)\psi^{*}\left(\frac{t-b}{a}\right)dt,$$

where C(a,b) represents the CWT coefficients, the symbol (*) denotes complex conjugation, ψ is the mother wavelet, *a* is the scaling parameter, and *b* represents the time-shifting parameter or translation of x(t). The coefficient matrix *C* obtained through the CWT is then converted into a time-frequency image. It is important to note, as mentioned in [48], that selecting the most suitable mother wavelet for specific problems can be a challenge. Different wavelet choices applied to the same signal can yield varying results. Additionally, the different scales and signal lengths are shifted throughout the entire dataset, and the results are multiplied by the sampling interval to obtain meaningful coefficients.

In [21], the Morlet wavelet is employed for non-stationary time series due to its effective auto-correlation performance and low cross-correlation characteristics. The Morlet wavelet function is defined as:

(3) $$\psi \left(t\right)=c_{\sigma }\pi^{-\frac{1}{4}}e^{-\frac{1}{2}t^{2}}\left(e^{i\sigma t}-e^{-\frac{1}{2}\sigma^{2}}\right),$$

where $c_{\sigma }$ is given by:

(4) $$c_{\sigma }={\left(1+e^{-\sigma^{2}}-2e^{-\frac{3}{4}\sigma^{2}}\right)}^{-\frac{1}{2}}.$$

**Figure 2:**
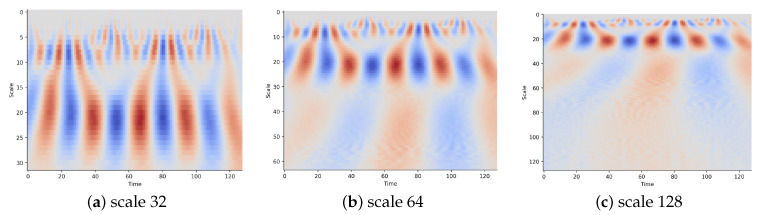
CWT images of a signal sequence at different scales.

To determine the appropriate parameters σ and *a* for constructing an optimal wavelet transform, we introduce an algorithm for automatically and adaptively selecting the wavelet scale in CWT. The details are shown in Algorithm 1. This algorithm consists of two procedures. The first procedure selects the optimal σ based on Shannon entropy, as described in [49]. Shannon entropy is used to assess the sparsity of wavelet coefficients. The corresponding shape factor σ is chosen to minimize Shannon entropy, resulting in wavelet transform coefficients with higher sparsity. The second procedure optimizes the selection of the scale parameter based on singular value decomposition (SVD), following the approach outlined in [50]. The goal is to obtain the maximum periodicity ratio δ using SVD on the coefficients matrix. This helps determine the optimal scale parameters for the wavelet transform. Figure 2 provides a visual representation of CWT images generated from a signal sequence at different scale parameters using the Morlet wavelet function. Generally, a broad range of scales can capture more information about slow changes, which can enhance classification accuracy.
**Algorithm 1** Optimal wavelet transform scale algorithm 1:**Input:** sensor signal x(t), σ = [$\sigma_{0}$,..,$\sigma_{k}$], *a* = [$a_{0}$,...,$a_{k}$] 2:Initial value $\sigma_{0}$, $a_{0}$                   ▹σ: shape factor, *a*: scale 3:**Procedure 1: Select σ with the minimal Shannon entropy** 4:**for** i=0,⋯,k **do** 5:      Compute CWT coefficients $C_{i}$(*a,b*) with size *m*×*n* 6:      Compute $d_{i}=\frac{c_{i,j}}{\sum_{1}^{m\times n}c_{i,j}}$      ▹$c_{i,j}$ is the element at the (i, j) position of the $C_{i}$ 7:      Compute Shannon entropy $H_{i}=-\sum_{i=1}^{m\times n}d_{i}\log d_{i}$ 8:      σ = agrmin$\left\{H_{i},H_{i+1}\right\}$ 9:**end for**10:**Procedure 2: Select *a* with the maximum periodicity**11:**for** j=0,⋯,k **do**12:      Compute CWT coefficients $C_{j}$13:      Compute SVD of matrix $C_{j}=UEV^{T}$▹$U^{T}U=I$, $V^{T}V=I$, E= diag$\left(\alpha_{1},\alpha_{2},\cdots ,\alpha_{p}\right)$, p=min(m,n)14:      Compute periodicity ratio $\delta_{j}={\left(\frac{\alpha_{i}}{\alpha_{i+1}}\right)}^{2}$15:      *a* = agrmax$\left\{\delta_{j},\delta_{j+1}\right\}$16:**end for**17:**Output:** (σ, *a*)

### 4.2. DCNN Architecture

We propose DCNN by exploiting the original CNN and adding the residual attention blocks (RAB). The DCNN architecture is shown as a part of Figure 1a. The proposed DCNN includes two convolutional (Conv) layers, two RAB as in Figure 1b, two max pooling (MP), and a fully connected (FC) layer. The details of the proposed DCNN model for the layer names and each layer’s hyperparameter settings are listed in Table 2.

#### 4.2.1. Convolutional Layer

CWT transforms sensor signal inputs into CWT images. The CWT images are denoted $x(q_{l},p_{l})$, where $q_{l}$ and $p_{l}$ represent the length and width of the time-frequency images, respectively. Then, CWT images are fed into the convolutional layer. The output $C_{ln}$ of convolutional layer is formulated as

(5) $$C_{ln}=f\left(x\cdot W+B\right)$$

where *W* and *B* represent weight and bias, respectively. Here, *f* represents the activation function of nonlinear mapping. The actual size of the feature image is denoted as

(6) $$S\left(C_{ln}\right)=\left[\frac{q_{l}+2\times r-q_{s}}{s}+1\right]\times \left[\frac{p_{l}+2\times r-p_{s}}{s}+1\right]\times K_{C},$$

where $K_{C}$ is the number of the convolution kernel, $q_{s}$ and $p_{s}$ are the length and width of the convolutional kernel, respectively. *r* is the edge extension parameter. *s* is the step size of the convolutional kernel.

#### 4.2.2. Residual Attention Block

The RAB serves as a crucial component of our proposed DCNN architecture, as depicted in Figure 1b. Each RAB consists of two convolutional layers and a residual connection, which can be represented as:

(7) $$H\left(x\right)=f\left(C_{ln}+x\right)$$

Here, H(·) and *x* denote the output and input of the RAB, respectively. $C_{ln}$ represents the output of the convolutional layer before the summation operation. The activation function *f* is defined as:

(8) f(x)=max(0,x)

Between the two convolutional layers, we apply batch normalization (Batch Norm) and ReLU activation. Batch Norm normalizes the outputs from the first hidden layer before passing them as inputs to the next hidden layer, improving convergence during training. Additionally, the residual connection facilitates the aggregation of low-level and high-level features in an additional way, addressing the issue of gradient vanishing that can occur in deep networks.

#### 4.2.3. Max Pooling Layer

The pooling layer reduces the dimensionality of output feature maps by replacing the output of a particular network position with the overall statistical characteristics of its neighboring outputs. The MP kernel is denoted as $P(q_{m}\times p_{m})$, where $q_{m}$ and $p_{m}$ represent the length and width of MP, respectively. The MP layer identifies multiple feature images $P_{i}$ as follows:

(9) $$P_{i}=max_{P(q_{m}\times p_{m})}C_{ln},$$

where $P_{i}$ ranges from 1 to $K_{p}$, and $C_{ln}$ has dimensions q×p. The output size of the MP layer is calculated as:

(10) $$S\left(P_{i}\right)=\left[\frac{q+2\times r-q_{m}}{s}+1\right]\times \left[\frac{p+2\times r-p_{m}}{s}+1\right]\times K_{p},$$

where $K_{p}$ represents the number of MP kernels.

#### 4.2.4. Fully Connected Layer

The output from the RAB layer is passed to the FC output layer with a softmax activation, which classifies the input into a given number of classes. In the FC layer, each neuron performs a linear transformation on the input vector using a weight matrix. This product is then passed through a non-linear activation function. Specifically, in this paper, the FC operation is defined as:

(11) Z=f(u×W+B).

Here, *u* represents the output from the previous layer, *Z* is the output of the FC layer, and *W* and *B* denote the weight and bias terms, respectively. The activation function *f* used is ReLU.

To classify the input data into their respective classes, a softmax activation function is employed at the final output layer. The softmax function takes a vector of FC layer outputs and returns a vector of probability scores. The equation for the softmax activation function is as follows:

(12) $$softmax{\left(z\right)}_{i}=\frac{e(z^{i})}{\sum_{j=1}^{N}e^{z_{j}}},$$

where *z* is the vector of FC layer outputs, *N* is the number of classes, and the *i*-th entry in the softmax output vector, softmax(z), represents the predicted probability of the test input belonging to class *i*.

### 4.3. Model Training

The DCNN architecture consists of two convolutional (Conv) layers, two residual attention blocks (RABs), two max pooling (MP) layers, and a fully connected (FC) layer. The DCNN learning procedure is presented in Algorithm 2. Forward propagation is conducted using Equations (4)–(11), where information flows from the input layer through the hidden layers to the output layer, resulting in the model’s output. Each forward propagation iteration produces the model’s error value. To calculate this error, we employ the cross-entropy cost function:

(13) $$L=-\frac{1}{n}\sum_{1}^{n}\left[y\log\hat{y}+\left(1-y\right)\log\left(1-\hat{y}\right)\right],$$

where *n* represents the number of training samples, $y_{i}$ is the actual label, and ${\hat{y}}_{i}$ is the predicted value of the model. To fine-tune the weight and bias parameters layer by layer, we employ a gradient descent algorithm, performing error backpropagation with Adam optimization [51].
**Algorithm 2** DCNN learning algorithm1:**Input:** spectrogram images returned by Algorithm 1, data splitted into training, validation, and testing sets2:**Output:** (optimal weights and biases of DCNN)3:**repeat**4:      **Forward Propagation:** Prediction of the label is calculated as (4)–(11)5:      **Backward Propagation:** Conduct backward propagation with Adam optimization6:**until** convergence

## 5. Experiments

### 5.1. Dataset

Several public HAR datasets including WISDM, UCI-HAR, Heterogeneous, PAMAP2, and UniMiB SHAR are used for our evaluation. The statistics of these datasets are described in Table 3. There are several differences between them such as the number of subjects, the number of activities, the number of samples, and the length of each sample. The WISDM and UniMiB SHAR datasets are recorded sensor signals through a triaxial accelerometer. UCI-HAR, PAMAP2, and Heterogeneous datasets are collected from more than two different types of sensors such as accelerometer (A), gyroscope (G), and magnetometer (M). Each type of sensor generates the 3D acceleration signals typically corresponding to the *x*-axis, *y*-axis, and *z*-axis. The details of the datasets are as follows:

- WISDM [32] is collected from 9 users wearing smartphones equipped with a three-axial accelerometer. Each user performs 6 types of low-level daily activities (walking, jogging, upstairs, downstairs, sitting, and standing). The data are composed of triaxial accelerometer signals collected at a sampling frequency of 20 Hz. The length of the sliding window is equal to 10 s and the overlap rate is set to 90%. Therefore, the whole WISDM dataset includes 10,981 samples.
- UCI-HAR [31] is collected by 30 volunteers wearing a smartphone to record the accelerometer, gyroscope, and magnetometer signals, performing 6 activities (walking, upstairs, downstairs, sitting, standing, and lying). The sensor signals were preprocessed by applying noise filters and then sampled in fixed-width sliding windows of 2.56 s and 50% overlap (128 readings/window).
- Heterogeneous [52] contained sensing data of accelerometer and gyroscope. It was collected from 9 users performing 6 activities (biking, sitting, standing, walking, stair up, and stair down). This dataset has been investigated in a large number of simple activities HAR. The key fact of the dataset is collected by 12 different smartphones and smartwatches, which increases the complexity of the task. The data are sampled at the frequency of 100 Hz.
- PAMAP2 [53] recorded signals of the accelerometer, gyroscope, magnetometer, temperature, and heart rate sensor. We select accelerometer, gyroscope, and magnetometer sensor signals for evaluation. The dataset is collected from 9 users while performing 12 activities (lying, standing, sitting, walking, cycling, nordic walking, ascending stairs, descending, running, ironing, vacuum cleaning, jumping rope).
- UniMiB SHAR [54] is recorded sensor signals through a triaxial accelerometer. It is performed by 30 participants along with 17 activities, including 9 different types of activities of daily living (StandingUpFL, LyingDownFS, StandingUpFS, Running, SittingDown, GoingDownS, GoingUpS, Walking, Jumping) and 8 different types of falls (Falling-BackSC, FallingBack, FallingWithPS, FallingForw, FallingLeft, FallingRight, HittingObstacle, Syncope). The window length and overlap rate are set to around 3 s and 50%, respectively. Data are sampled at a frequency of 50 Hz, which provides 11,771 acceleration samples.

**Table 3:** Description of datasets.

Dataset	Subject	Sample Rate	Activity	Length	Sensor	Sample
WISDM [32]	29	20	6	200	A	10,981
UCI-HAR [31]	30	50	6	128	A, G	10,470
Heterogeneous [52]	9	100	6	128	A, G	43,930,257
PAMAP2 [53]	9	100	12	128	A, G, M	2,844,868
UniMiB SHAR [54]	30	50	17	151	A	11,711

Note: A is denoted accelerometer, G is denoted gyroscope, M is denote magnetometer.

### 5.2. Experimental Setup

In this paper, the experiment is run on a computer with an Intel Core i7 processor (Intel Corporation: Santa Clara, CA, USA) 16 GB of RAM. In terms of software, the Google COLAB server is used to compile the experimental analyses. Keras as a Python 3 library is used for conducting DL model training and parameter inference with automatic differentiation. In addition, dataset and results analysis is conducted by Matplotlib and Seaborn library.

As the architecture of DWCNN in Figure 1 is shown in Table 2, the training parameters of DWCNN and the other algorithms were first determined, as shown in Table 4. First, CWT is applied to sensor signal input to get CWT images and resize all with size 64 × 64. The kernel size of the convolutional layer is set to 5 × 5. In RAB, the size of the convolutional kernel is set to 3 × 3. The activation function is ReLU. The size of the max pooling window is 2 × 2. The size of the output FC layer is equal to the number of labels of each dataset. The DWCNN optimizes the scale parameter with a scale of 64, and DCNN optimizes by the Adam optimizer for the optimization of the cross-entropy loss function with a learning rate of 0.0001 with a batch size of 128, with training epochs of 100. The datasets are divided into a 70 % training set, a 10% validation set, and a 20% testing set. Furthermore, we run 10 repetitions of the experiments and report averaged measures as the final measures of a model’s performance.

### 5.3. Evaluation Measures

For the classification task, we use evaluation metrics such as accuracy (Acc), precision (P), recall (R), and F1 as shown in Equations (14)–(17).

(14) $$Acc=\frac{TP+TN}{TP+FN+TN+FP}$$

(15) $$P=\frac{TP}{TP+FP}$$

(16) $$R=\frac{TP}{TP+FN}$$

(17) $$F1=\frac{2\times P\times R}{P+R}$$

Here, TP and TN are the true positives and true negatives, and *FP* and FN are the false positives and false negatives, respectively. Precision is the ratio of correctly predicted positive observations to the total predicted positive observations. Recall is the ratio of correctly expected positive observations to all observations in the actual class. F1 score is a harmonic average of the P and R values. When there is an unbalanced distribution of classes, this measure is crucial.

### 5.4. Compared Methods

We compare DWCNN with the baseline and state-of-the-art methods. Firstly, the simple DNN methods on the time domain, which use only raw signal inputs without processing signals, such as CNN [55], LSTM [56], and CNN-LSTM [19]. Secondly, the hybrid DNN methods on the time domain or time-frequency domain with attention mechanism as state-of-the-art for HAR, specifically, such as DeepSense [57], and DanHAR [24] on the time domain, on the time-frequency domain such as AttnSense [18], and the CWT-DenseNet methods [21]. Finally, to verify the contributions of different components in the proposed DWCNN method, we consider two variants of the DWCNN model such as DWCNN-noCWT and DWCNN-noRAB. The details of compared methods are as follows:

- CNN [55]: A CNN model with three convolution layers, a pooling layer, and a fully connected layer.
- LSTM [56]: A simple LSTM model for time-series dataset.
- CNN-LSTM [19]: The model uses CNN to extract features and LSTM to learn time dependencies.
- DeepSense [57]: The model uses CNN to extract features of each sensor and combine them by another merge convolutional layer, then it uses LSTM to learn time dependencies.
- DanHAR [24]: The model presents residual networks with CNN and attention mechanisms to improve feature representation ability.
- AttnSense [18]: The model combines an attention mechanism with CNN and an improved LSTM to capture the dependencies of sensing signals in both spatial and temporal domains. The raw sensor signal inputs are required to transform into spectrograms as images by FFT.
- CWT-DenseNet [21]: The pre-trained model combines CWT and DenseNet [44] to extract features on the time-frequency domain.
- DWCNN-noCWT: A variant of DWCNN removes the CWT layer and only uses the proposed DCNN.
- DWCNN-noRAB: A variant of DWCNN removes the RAB blocks instead of convolution layers.

We compare DWCNN with the baseline and the state-of-the-art methods that verify the performance of our approach on the time-frequency domain based on hybrid wavelet transform and residual CNN-based techniques. We compare the simple and hybrid DNN methods to identify the effective proposed method on the time-frequency domain using CWT and the robustness of the DCNN architecture. We compare two variants of DWCNN to estimate potential implications of removing CWT layer or RAB blocks on the model’s performance. The role of each component is evaluated in DWCNN.

### 5.5. Results

**Figure 3:**
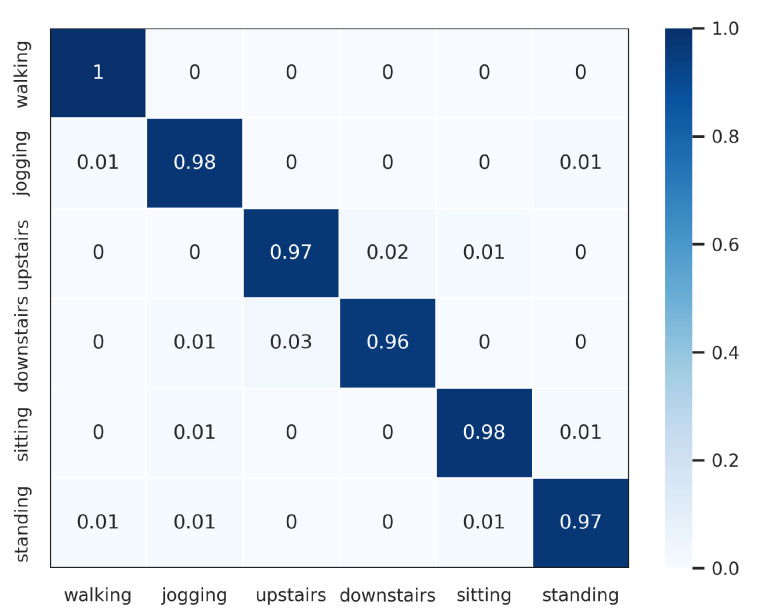
Confusion matrix of WISDM.

**Figure 4:**
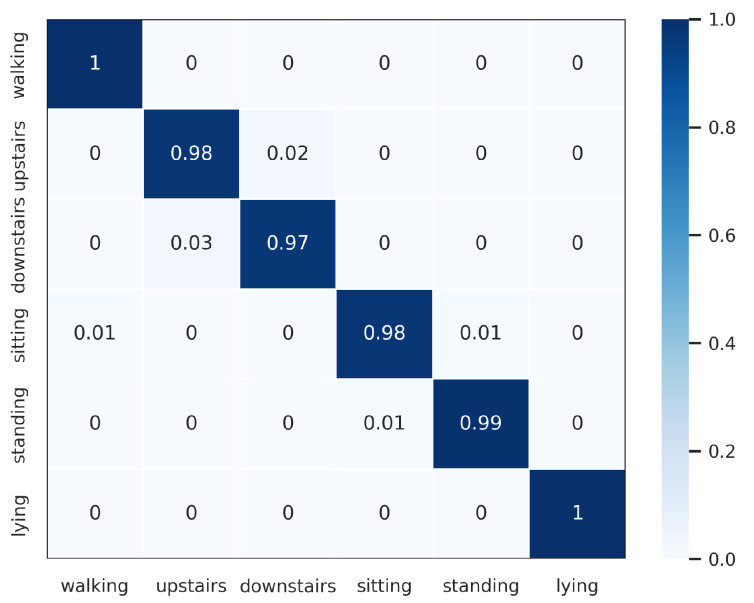
Confusion matrix of UCI-HAR.

**Figure 5:**
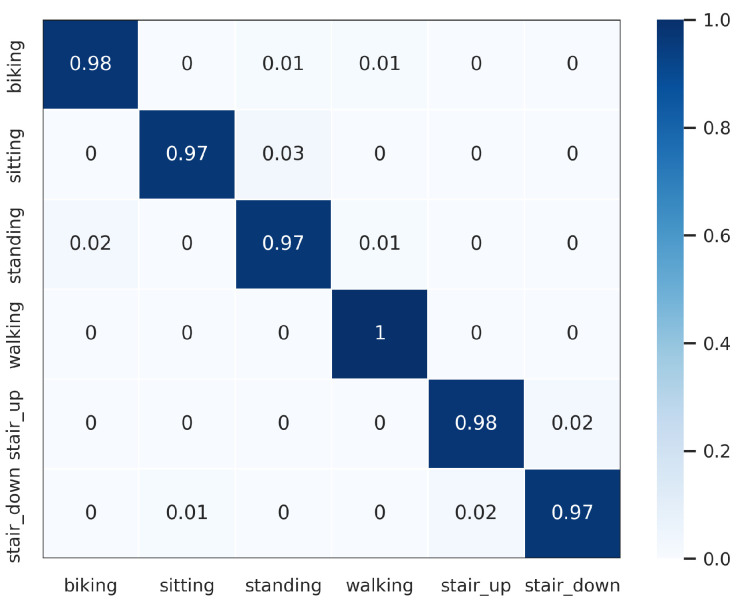
Confusion matrix of Heterogeneous.

**Figure 6:**
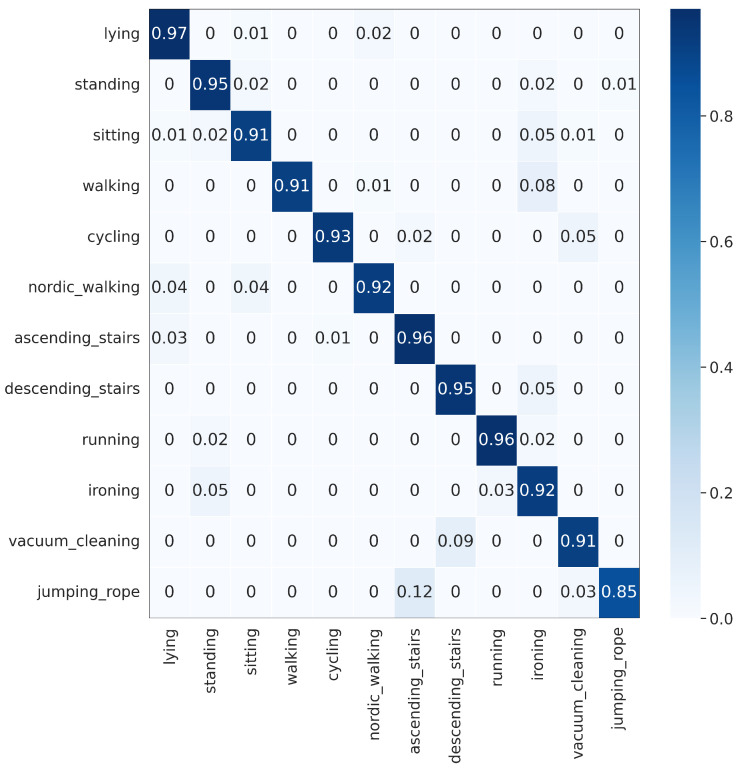
Confusion matrix of PAMAP2.

**Figure 7:**
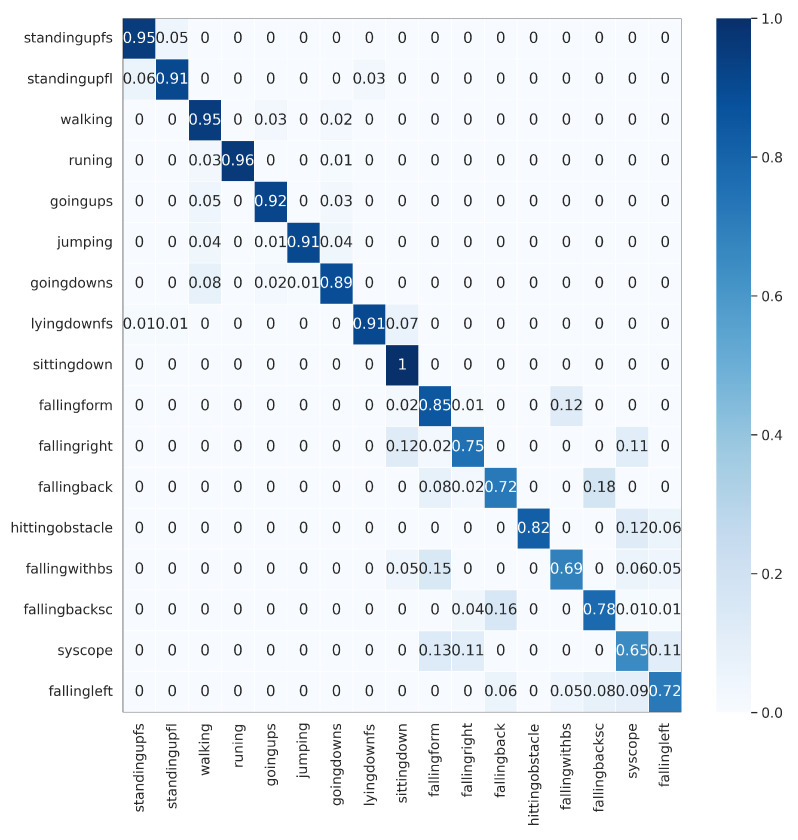
Confusion matrix of UniMiB SHAR.

The proposed DWCNN method was compared with the baseline and the state-of-the-art methods on five public datasets with the average F1 measure. The results are shown in Table 5, and the normalized confusion matrixes are illustrated in Figure 3, Figure 4, Figure 5, Figure 6 and Figure 7. Results demonstrate that DWCNN performs the best among all compared methods on all datasets.

DWCNN’s performance is better than the simple DNN methods such as CNN, LSTM, and CNN-LSTM, averaging from 8% to 15%. It verifies that the DWCNN has a greater capability to capture features in the time-frequency domain in multimodal sensing data for HAR. CNN, LSTM, and CNN-LSTM cannot capture features to distinguish between complex activities on the time domain because of the simple DNN architecture. The experiment results demonstrate that the spectrogram images provide more complementary information of signals by extracting the DCNN based on adding RABs. Therefore, the signal processing by CWT in DWCNN brings a large benefit instead of using raw signals for the HAR problem.

DWCNN’s performance is higher than the hybrid DNN methods on the time domain such as DeepSense and averaging from 2% to 5% on the WISDM, UCI-HAR, and Heterogeneous datasets and from 7% to 8% on the PAMAP2 and UniMiB SHAR datasets, respectively. In comparison with the hybrid DNN methods on the time-frequency domain, DWCNN’s performance is better than AttnSense and CWT-DenseNet methods averaging from 2% to 4% on WISDM, UCI-HAR, and Heterogeneous datasets and from 5% to 7% on PAMAP2 and UniMiB SHAR datasets, respectively. Although AttnSense and CWT-DenseNet can capture the dependencies of multimodal sensing signals in both spatial and temporal domains, they are not well enough for complex discrimination activities on the time-frequency domain on PAMAP2, and UniMiB SHAR datasets. The reason may be the different signal transform functions between the FFT of AttnSense and CWT of CWT-DenseNet. A limitation of the Fourier transform can only capture global frequency information over an entire signal. Therefore, the signal decomposition of the AttnSense method may not serve all HAR applications well where signals have short intervals of characteristic oscillation, whereas the CWT of CWT-DenseNet can decompose a signal directly according to the frequency and represent it in the frequency domain distribution state in the time domain. So, the signal’s time and frequency information are retained. The CWT-DenseNet combines Morlet wavelet with 256 scale values and DenseNet architecture with 121 trainable layers. However, the different wavelet functions and scale parameters of CWT in CWT-DenseNet are very complex. It is not effective for various activity recognition such as PAMAP2, and UniMiB SHAR datasets by 256 scale parameters with the complex CNN architecture. The key distinction of DWCNN on the compared methods is signal processing by auto sale parameter optimization with the Morlet wavelet function. To obtain meaningful and rich features by scale parameter and DNN optimization, the proposed DWCNN method uses the CWT algorithm to transform signals into spectrograms as images with auto-scale parameters. Then, the images are fed into the DCNN, which includes RABs to enhance capturing and extracting feature representation.

We employ the experiment cases with the two variants of DWCNN such as DWCNN-noCWT, and DWCNN-noRAB. The performance of DWCNN outperforms its variants. The DWCNN’s performance is higher than DWCNN-noCWT from 2% to 9% on the average F1 score. The F1 result of the proposed method is higher than DWCNN-noRAB from 1% to 5%. The performance of DWCNN-noRAB is higher than DWCNN-noCWT on the F1 score with five public datasets. These results emphasize the significance of signal transformation by CWT in our proposed method. The key reason is signal processing by CWT with auto-optimal scale parameter selection based on Algorithm 1 to extract the distinguish spectrograms such as images. Consequently, the averaging F1 results of DWCNN-noRAB on the time-frequency domain are better than DWCNN-noCWT on the time domain on all datasets. However, the results imply that DWCNN-noRAB cannot defeat DWCNN. The main reason is residual connections in RABs in DCNN of DWCNN that address the generalization ability of DNNs. Therefore, DCNN can extract distinctive features from simple and complex activities. Based on hybrid signal transformation by CWT and DCNN, the DWCNN model is an effective and robust method for multimodal wearable sensor-based HAR problems.

In order to further verify the outperforming of the proposed method for each class label on each HAR dataset, we normalized confusion matrixes, which are shown in Figure 3, Figure 4, Figure 5, Figure 6 and Figure 7. The confusion matrix of three datasets such as WISDM, UCI-HAR, and Heterogeneous, is composed of six activities in Figure 3, Figure 4 and Figure 5, respectively. The confusion matrixes show that our proposed method performs well in distinguishing six simple activities. Specifically, the performance of six simple activities on three datasets such as WISDM, UCI-HAR, and Heterogeneous outperforms by 95% to 99% accuracy measure. In several specific cases, the accuracy performance of walking activity is 100%. Figure 6 and Figure 7 show the confusion matrix of PAMAP2 and UniMiB SHAR that DWCNN is effective well on both simple and complex activities. The reason is that DWCNN can leverage complementary information from both spatial and temporal domains to compute more discriminative complex activities. Therefore, DWCNN can improve the performance of HAR classification. However, several class labels of UniMiB SHAR are only higher than about 70% (such as syscope, fallingleft, fallingbacksc, fallingwithbs, fallingback, and fallingright). The main reason may be their interfering class labels and the number of samples for their class labels is not enough for distinguished learning.

**Figure 8:**
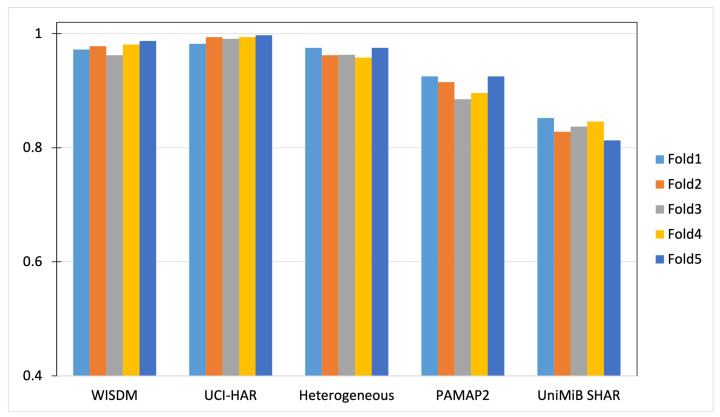
Accuracy results of five-fold cross-validation on five datasets.

We perform a five-fold cross-validation for all datasets to evaluate the generality of the proposed DWCNN method. The five-fold cross-validation processing is performed as follows. Each dataset is randomly partitioned into five folds, in which each fold is held out in turn and the training is performed on the rest four-fifths. Thus, the learning processing is executed overall five times on different training sets. The accuracy results are shown in Figure 8. The performance of DWCNN on three datasets such as WISDM, UCI-HAR, and Heterogeneous, obtains more than a 97% accuracy measure. The performance of DWCNN on PAMAP2 has more than a 92% accuracy measure. On UniMiB SHAR, accuracy is more than 82%. The performance of DWCNN on WISDM, UCI-HAR, and Heterogeneous is higher than PAMAP2 and UniMiB SHAR. Several significant reasons are the number of class labels, the type of activities (simple activity, complex activity), and the ratio between the number of class labels with total samples. Specifically, three datasets such as WISDM, UCI-HAR, and Heterogeneous have six class labels with simple activities, whereas PAMAP2 and UniMiB SHAR have twelve and seventeen class labels including simple and complex activities, respectively. UniMiB SHAR has a large number of class labels but the number of samples for each class label is small. Therefore, UniMiB SHAR does not have enough samples for the training model to distinguish activities.

### 5.6. Ablation Experiments

**Figure 9:**
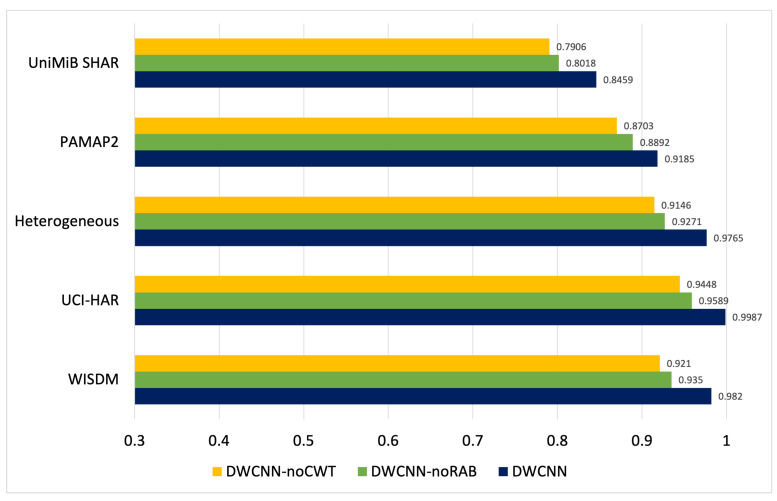
Accuracy results of three models on five datasets.

Ablation experiments with two models such as DWCNN-noCWT and DWCNN-noRAB are examined to thoroughly evaluate the suggested DWCNN model. The first method, DWCNN-noCWT, is implemented to test the effectiveness of the learning original signal by combining RABs with CNN without CWT. The second method, DWCNN-noRAB, evaluates the effectiveness of learning CWT and CNN models without RAB. The accuracy results of comparing three models on five datasets are shown in Figure 9. The DWCNN performance outperforms the two compared variants (DWCNN-noCWT and DWCNN-noRAB). The DWCNN performance is higher than DWCNN-noCWT by about 5%, 4%, 6%, 5% and 6% on UniMiB SHAR, PAMAP2, Heterogeneous, UCI-HAR, WISDM, respectively. This indicates that the CWT phase in DWCNN has a significant role in the DNN. Based on CWT, the original sensor signals were processed from the time domain into the time-frequency domain with meaningful spectrogram images. In addition, combining RAB and CNN architecture in the proposed method is suitable for spectrum images. The DWCNN performance is higher than DWCNN-noRAB by about 4%, 3%, 4%, 4% and 5% on UniMiB SHAR, PAMAP2, Heterogeneous, UCI-HAR, WISDM, respectively. Specifically, on the WISDW dataset, the DWCNN-noCWT, and DWCNN-noRAB have a classification accuracy of 92.16% and 93.59%, respectively, while the DWCNN acquires a better result of 98.26%. With UCI-HAR, the DWCNN-noCWT, DWCNN-noRAB, and DWCNN have a classification accuracy of 94.48%, 95.58%, and 99.87%, respectively. The accuracy results of DWCNN-noCWT, DWCNN-noRAB, and DWCNN models are 91.46%, 92.71%, and 97.65% on the Heterogeneous dataset. The accuracy results of DWCNN-noCWT, DWCNN-noRAB, and DWCNN models are 87.03%, 88.92%, and 91.85% on the PAMAP2 dataset. On the UniMiB SHAR, the accuracy of DWCNN-noCWT, DWCNN-noRAB, and DWCNN models are 79.06%, 80.18%, and 84.59%, respectively.

The performance of DWCNN outperforms its variants. The accuracy result of DWCNN-noRAB is higher than DWCNN-noCWT on five public datasets. These results emphasize the significance of signal transformation by CWT in our proposed method. The key reason is signal processing by CWT with auto-optimal scale parameter selection based on Algorithm 1 to extract the distinguish spectrograms such as images. Consequently, the accuracy results of DWCNN-noRAB on the time-frequency domain are better than DWCNN-noCWT on the time domain on all datasets. In addition, the results imply that DWCNN-noRAB cannot defeat DWCNN. The main reason is residual connections in RABs in DCNN of DWCNN that address the generalization ability of DNNs. Therefore, DCNN can extract distinctive features from simple and complex activities. Based on hybrid signal transformation by CWT and DCNN, the DWCNN model is an effective and robust method for multimodal wearable sensor-based HAR problems.

**Figure 10:**
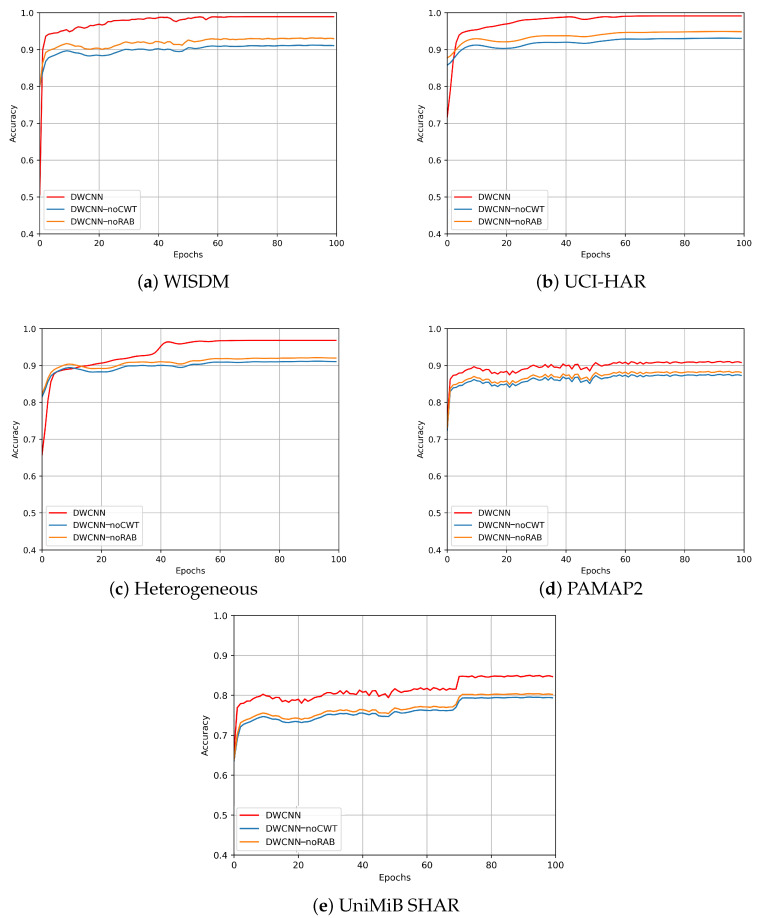
Performance comparisons on five public datasets on accuracy measure.

In order to evaluate the effectiveness of the proposed method with two variant methods, the number of training epochs is constantly set to 100 during the whole experiment. As accuracy results following epochs from five public datasets such as WISDM, UCI-HAR, Heterogeneous, PAMAP2, and UniMiB SHAR, respectively, such as Figure 10a–e, the DWCNN model’s performances outperforms DWCNN-noCWT and DWCNN-noRAB models. Therefore, the CWT phase and RAB significantly contribute to the performance gain when compared with two variant baselines across five public datasets. Especially, there is an increase of about 4% and 6%, respectively, over the DWCNN-noRAB and DWCNN-noCWT models on all datasets.

**Figure 11:**
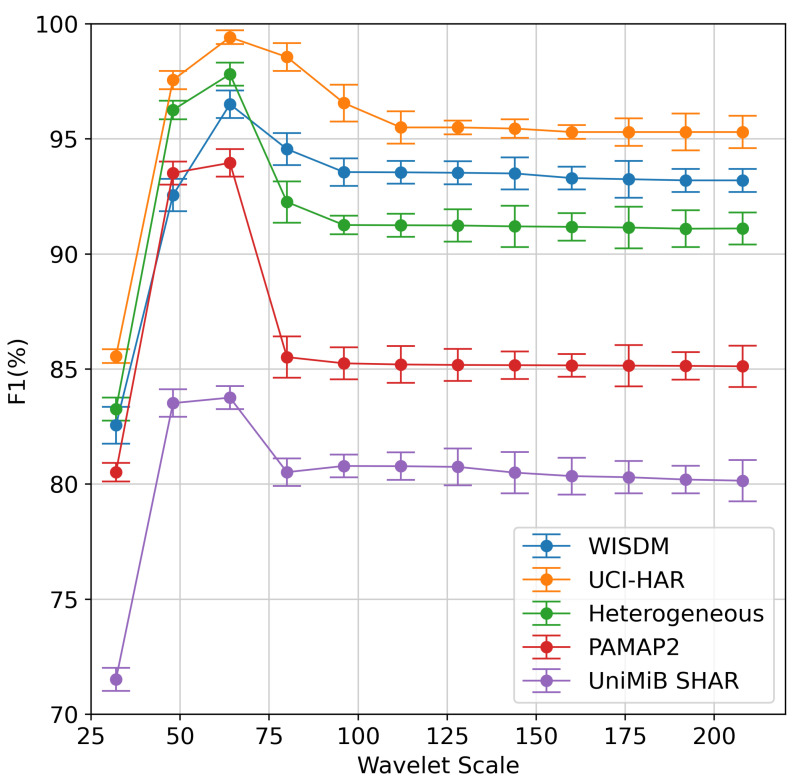
The performance of HAR under different wavelet scales.

We implement our proposed method on five datasets with different scale parameters to evaluate the effectiveness of finding the scale optimization parameter in Algorithm 1. The averaging F1 results are given in Figure 11. The results show that the best number of wavelet scale parameters for both datasets is 64. It can be found that increasing the scale size from 32 to 64 tends to improve the performance of the model. When the scale size is more than 64, the model’s performance decreases and tends to stabilize after a certain scale size. The reason is that CWT can be able to extract time-frequency information. The smaller scales, such as $2^{0}$, $2^{1}$, and $2^{2}$, correspond to high frequencies and thus predominantly consist of noise in raw signals. When we go up in scale (i.e., in $2^{5}$, $2^{6}$), we observe bright light corresponding to the activity. However, we can lose the signal in the larger-scale coefficients (i.e., more than $2^{6}$), which are associated with low-frequency information.

**Figure 12:**
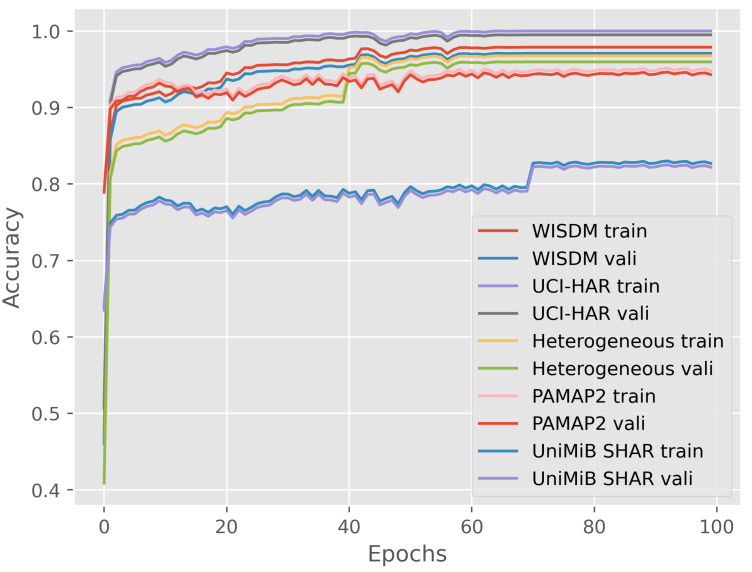
Accuracy change with epochs during training.

We investigate the number of training epochs to give the proposed model enough space. Figure 12 shows the training and validation accuracy along training epochs on five datasets. Accuracy is calculated as the number of correct predictions divided by the total number of predictions made by the model. Reviewing the learning curves, we can see that the model of the proposed method converges well with performance on the validation dataset at around 40 epochs with WISDM, UCI-HAR, Heterogeneous, and PAMAP2 datasets. The improvement trend continues with the UniMiB SHAR at around 70 epochs. The results imply that the number of training epochs of the DWCNN model is small to obtain effective performance on all datasets. Therefore, DWCNN is a generality architecture model for multimodal sensor-based HAR.

## 6. Conclusions

Human activity recognition from multimodal sensing data is a challenging task. In this paper, we propose the DWCNN method to learn features from the time-frequency domain and improve accuracy for the HAR task by combining scale parameter optimization in the CWT algorithm and RABs in DCNN architecture. As demonstrated in the experiments on five public HAR datasets, the proposed method outperforms baseline and state-of-the-art methods with WISDM, UCI-HAR, Heterogeneous, PAMAP2, and UniMiB SHAR datasets in terms of 98.56%, 99.56%, 97.26%, 93.52%, and 83.52% F1 scores, respectively. The proposed DWCNN significantly enhanced the performance of multimodal HAR since the proposed method can automatically learn features from the time-frequency domain based on the hybrid CWT and DCNN model.

In real-world situations, wearable-based systems can have problems with the loss of sensor signals or noisy data, so combining the different modalities may resolve their limitations and provide better solutions. Using vision and wearable sensors for HAR can solve some limitations and be used for healthcare applications. In future work, we would explore the fusion of vision and wearable sensors that may provide view-invariant features, which will be more useful in a realistic environment.

## Figures and Tables

**Table 1:** Descriptions of related works.

Refers	Method	Domain	Detail	Limitation
Dua et al. 2021 [42]	CNN-GRU	Time	It achieves superior classification performance based on hybrid CNN and GRU	Model is hard to train and high computation cost.
Gao et al. 2021 [24]	DanHAR	Time	Model presents residual networks and attention mechanisms to improve feature representation ability.	Model needs to more labeled multimodal samples for the decisions of neural models.
Khatun et al. 2022 [19]	CNN-LSTM	Time	The hybrid DL models use the self-attention algorithm to enhance the predictive capabilities of the system. CNN is used for spatial feature extraction, and the LSTM network is utilized for learning temporal information.	It is not able to perform better in the case of multiple people and more complex physical activities.
Lee et al. 2023 [20]	MCNN	Time	It effectively extracts features using multiple CNNs with different kernel sizes and an attention layer to each channel and spatial level.	It performs poorly and needs to add a transform layer for the specific domain.
Yan et al. 2023 [40]	Spatial-Temporal Graph CNNs	Time	It offers an outstanding opportunity for fall detection by extracting the motion features of human falls and the activities of daily living (ADL s) at the spatial and temporal scales for fall detection.	It is more influenced by window size for types of falls.
Ma et al. 2019 [18]	AttnSense	Time-frequency	The model capture the dependencies of sensing signals in prioritized sensor selection and improves the comprehensibility	fast Fourier transform (FFT) tendency to capture global frequency information over the entire signal.
Liu et al. 2023 [29]	MAG-Res2Net	Time	Model used directly two DL architectures Res2Net [30] with adding the gated module to improve performance of multimodal HAR.	Nevertheless, certain proposed techniques like Loss Combined and multi-scale networks increase computational or memory costs.
Dahou et al. 2023 [36]	MLCNNwav	Time-frequency	Model relies on residual CNNs and then one-dimensional trainable DWT.	Complex transform the output of MLCNNwav by Daubechies wavelet family with coefficients ranging from 1 to 6.
Showmik et al. 2023 [37]	PCWCNN	Time-frequency	Model uses principal component analysis and the DWT from signal feed into residual CNNs.	Time-consuming and complex in the step signal processing for reducing noise since typical frequency domain filters.
Pavliuk et al. 2023 [21]	CWT-DenseNet	Time-frequency	The pre-trained model combines different CWT configurations and DenseNet [44] with 121 trainable layers to improve the accuracy of HAR performance.	Select scale parameters and CWT functions cost time and complex residual CNNs-based architecture.

**Table 2:** Description of hyperparameter settings.

Layer Name	Hyperparameter Settings
Conv	Activation = ReLU, Strides = 1, Kernel Size = 5
RAB	2 Conv: Activation = ReLU, Strides = 1, Kernel Size = 3, Batch Norm
Conv	Activation = ReLU, Strides = 1, Kernel Size = 5
MP	Padding = Same, Strides = 1, Pool Size = 2
RAB	2 Conv: Activation = ReLU, Strides = 1, Kernel Size = 3, Batch Norm
MP	Padding = Same, Strides = 1, Pool Size = 2
FC	Activation = Softmax

**Table 4:** Values of the training parameters of DWCNN.

Parameter	Value
Scale_parameter	32, 64, 128
Wavelet_function	Morlet wavelet
Minibatch_size	128
Activation_function	ReLU
Batch_Normalization	−1
Learning_rate	0.0001
Max_epochs	100
Optimizer_function	Adam
Classifier_function	Softmax
Loss	Cross-Entropy function

**Table 5:** Comparison of the DWCNN with the compared methods on average F1 measure and standard deviation.

Method	WISDM	UCI-HAR	Heterogeneous	PAMAP2	UniMiB SHAR
CNN [55]	0.8580 ±0.0346	0.9024 ±0.0312	0.8080 ±0.0291	0.8170 ±0.0361	0.7483 ±0.0415
LSTM [56]	0.8020 ±0.0289	0.8591 ±0.0318	0.8120 ±0.0331	0.7510 ±0.0297	0.7521 ±0.0367
CNN-LSTM [19]	0.8706 ±0.0158	0.9134 ±0.0293	0.8560 ±0.0192	0.7480 ±0.0235	0.7739 ±0.0314
DeepSense [57]	0.8605 ±0.0179	0.9218 ±0.0136	0.9310 ±0.0256	0.8250 ±0.0278	0.7602 ±0.0286
DanHAR [24]	0.9795 ±0.0313	0.9742 ±0.0245	0.9724 ±0.0213	0.9316 ±0.0256	0.7903 ±0.0356
AttnSense [18]	0.9520 ±0.0296	0.9645 ±0.0327	0.9650 ±0.0101	0.8930 ±0.0134	0.7712 ±0.0256
CWT-DenseNet [21]	0.9456 ±0.0192	0.9517 ±0.0267	0.9127 ±0.0145	0.8802 ±0.0254	0.8216 ±0.0219
DWCNN-noCWT	0.9136 ±0.0216	0.9056 ±0.0387	0.9015 ±0.0218	0.9137 ±0.0357	0.8126 ±0.0238
DWCNN-noRAB	0.9649 ±0.0231	0.9418 ±0.0215	0.9437 ±0.0156	0.9216 ±0.0286	0.8245 ±0.0225
**DWCNN**	**0.9856** **±0.0121**	**0.9956** **±0.0156**	**0.9726** **±0.0225**	**0.9352** **±0.0197**	**0.8352** **±0.0183**

## Data Availability

Data are contained within the article.
